# Mesoporous Titanium Dioxide: Synthesis and Applications in Photocatalysis, Energy and Biology

**DOI:** 10.3390/ma11101910

**Published:** 2018-10-09

**Authors:** Ben Niu, Xin Wang, Kai Wu, Xianru He, Rui Zhang

**Affiliations:** 1 School of Materials Science and Engineering, Energy Polymer Research Center, Southwest Petroleum University, 8 Xindu Avenue, Chengdu 610500, China; 201721000054@stu.swpu.edu.cn (B.N.); 201531053327@stu.swpu.edu.cn (K.W.); 2 Institute für Physik, Universität Rostock, Albert-Einstein-Str. 23–24, 18051 Rostock, Germany

**Keywords:** mesoporous titanium dioxide, hybrid material, synthesis, application

## Abstract

Mesoporous materials are materials with high surface area and intrinsic porosity, and therefore have attracted great research interest due to these unique structures. Mesoporous titanium dioxide (TiO_2_) is one of the most widely studied mesoporous materials given its special characters and enormous applications. In this article, we highlight the significant work on mesoporous TiO_2_ including syntheses and applications, particularly in the field of photocatalysis, energy and biology. Different synthesis methods of mesoporous TiO_2_—including sol–gel, hydrothermal, solvothermal method, and other template methods—are covered and compared. The applications in photocatalysis, new energy batteries and in biological fields are demonstrated. New research directions and significant challenges of mesoporous TiO_2_ are also discussed.

## 1. Introduction

Mesoporous materials are of great importance for their unique structure and properties, thus finding many potential applications in catalysis, biological medicine and environmental energy. Since the Mobil Oil Corporation discovered ordered mesoporous silica in 1992 [1,2], the synthesis and applications of mesoporous materials have been broadly studied. In 1995, mesoporous TiO_2_ was first synthesized by Antonelli and Ying with modified sol–gel method [3]. Since then, mesoporous TiO_2_ has attracted more and more interest due to its great advantages, including good chemical and physical properties, non-toxicity and good biocompatibility, excellent photoelectric performance, as well as their broad applications in photocatalysis, energy and biology. The mesoporous TiO_2_ materials can be classified into disordered and ordered structure according to the arrangement of pores in space. For the disordered mesoporous TiO_2_, the pores formed by the accumulation between particles and particles are irregular and not interconnected, and the pore size distribution is wide; Whereas, for ordered mesoporous materials, the pores are regularly arranged in space and the pore size distribution is narrow. Over the past decades, various mesoporous TiO_2_ with different morphologies, surface areas and pore volumes had been synthesized and applied in many fields [4,5,6].

In recent years, there have been some excellent reviews regarding mesoporous TiO_2_ in various aspects [7,8,9,10,11,12,13,14,15,16,17]. Different synthesis methods, morphologies and pore-wall parameters of mesoporous TiO_2_ were summarized and discussed. Meanwhile, applications in photocatalysis, new energy (such as solar cells and lithium-ion batteries) as well as in biological fields have raised more attention hence further helped push forward the development of this type of material. This review focuses on the most recent study of the synthesis, morphology, doping and crystallization of mesoporous TiO_2_ that are closely related to applications in photocatalysis, energy and biology. Particular emphases are given to the latest achievements in the synthesis of mesoporous TiO_2_ as well as the relationship to the structure.

## 2. Synthesis

The synthesis methods of mesoporous TiO_2_ materials have been widely studied in the past two decades, whether it is for new applications where specific morphology is required, or simply for better control of morphology and/or improvement of reaction including sol–gel, hydrothermal, solvothermal and template reaction. A fabrication of mesoporous TiO_2_ usually includes the construction of mesoporous spaces (2–50 nm) and arranging them in assembled arrays. The commonly used synthesis methods include sol–gel, hydrothermal and solvothermal methods, and template methods. Different synthesis approaches give rise to different morphologies, mesostructures, pore sizes, doping and crystallization of mesoporous TiO_2_ materials. These characteristics are structurally important for the applications. In this section, different synthesis methods are briefly revisited and compared, mesoporous TiO_2_ with these characteristics is also discussed.

### 2.1. Sol–Gel Method

**Figure 1:**
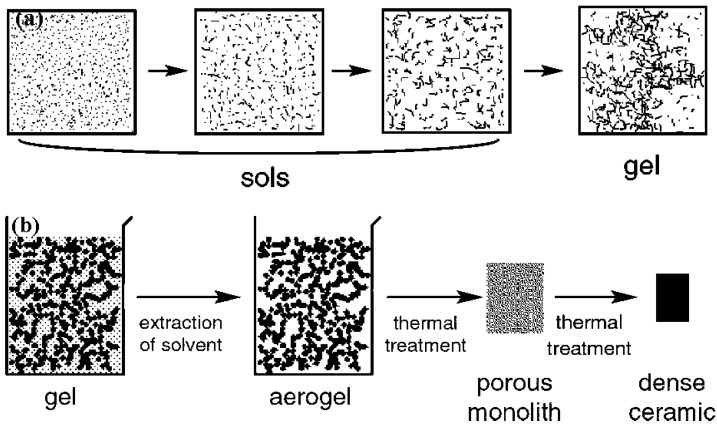
(**a**) The process of sol–gel transition. (**b**) Drying and calcining the gel to get molecular and nanostructured materials. Reprinted with permission from [18]. Copyright 2018, American Chemical Society.

Nowadays, the sol–gel method is the most common and versatile way for preparing mesoporous TiO_2_, because it is easier to carry out than as in the solid state, and only requires a low synthesis temperature. Besides, it can prepare a variety of new mesoporous TiO_2_ materials by selecting the appropriate conditions. However, sol–gel approach usually takes days or weeks to finish, thus it is much slower in the time sense. There is a transition of the system from liquid solution (Sol) to solid gel phase (Gel) in the sol–gel process. First, a gel is obtained by a colloidal suspension of solid particles in a liquid. Then the sol slowly polymerizes between the particles to form a three-dimensional gel network structure, and gel networks are filled with a loss of mobility of the solvent, finally the gel is formed (Figure 1a). Next, the gel is dried and calcined to get molecular or even nanostructured materials (Figure 1b) [18]. In the 1960s, sol–gel method was developed for synthesizing various materials including all kinds of mesoporous materials [9]. These mesoporous materials, which have high specific surface area, narrow pore size distribution and adjustable pore size, are mainly classified into silicon-based mesoporous materials (SiC, SiCN, SiOC, etc.) and non-silicon-based mesoporous materials (transition metal oxides, sulfides, carbides, nitrides, etc.) Meanwhile, the preparation of mesoporous TiO_2_ has attracted a wide range of interests.

For instance, Liu and coworkers prepared mesoporous TiO_2_ with anatase structure via sol–gel reaction [19]. It was found that the products have specific surface area, total pore volume and average pore size with 106 m^2^·g^−1^, 0.17 cm^3^·g^−1^ and 4.8 nm, respectively. This synthesis approach provides an environmentally-friendly pathway, which is of great significance for the applications of mesoporous TiO_2_.

The sol–gel approach to synthesize mesoporous TiO_2_ generally includes the acids/bases-catalyzed hydrolysis and condensation step of Ti precursor. Therefore, the presence of acids and bases have a significant influence on the surface area and pore parameters of TiO_2_. Claudia et al. used H_3_PO_4_ as the hydrolysis catalyst and anionic precursor to prepare TiO_2_ using sol–gel method [20]. TiO_2_ with higher surface area and smaller crystallite size was obtained due to the existence of phosphate anion (Table 1). The phosphate anion can be easily adsorbed on the surface of TiO_2_, so the material negatively charged hence has a strong tendency to form hydrogen bonds. In addition to these conventional sol–gel methods, an approach without acids or bases was also reported [21]. The product is an anatase phase with pure and highly crystalline, very high porosity and a large surface area of 226.25 m^2^·g^−1^.

**Figure 2:**
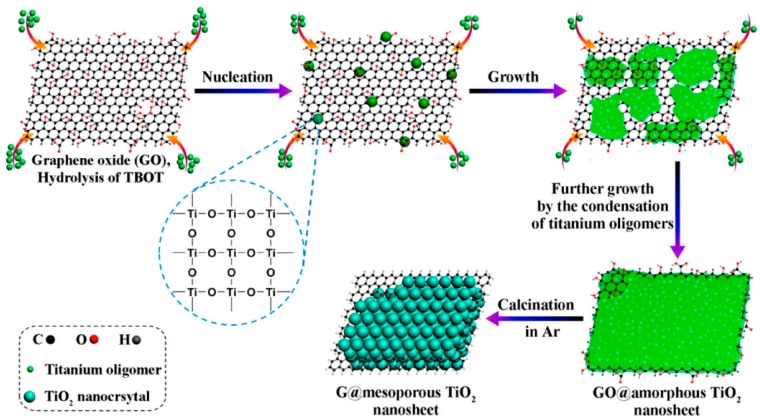
Schematic formation process of the mesoporous TiO_2_/graphene/mesoporous TiO_2_ sandwich-like nanosheets. Reprinted with permission from [22]. Copyright 2018, American Chemical Society.

Mesoporous TiO_2_ prepared by sol–gel method is usually disordered and generally exhibits a wormhole-like mesostructure rather than a large range of ordered regularity. This is due to the highly reactive Ti precursors, and their hydrolysis and condensation rates which are often not easy to control during the sol–gel process. Attempts leading to more ordered TiO_2_ were continuously made by researchers. For example, Zhao et al. recently synthesized a uniform mesoporous TiO_2_ material involving graphene oxide (GO), where the GO sheet is served as the substrate for the growth of TiO_2_ [22]. During the synthesis (Figure 2), GO sheets were coated with amorphous TiO_2_ shells, and the product (G@mTiO_2_) was obtained through calcination by controlling the hydrolysis and condensation of the Ti precursors. The as-prepared sandwich-like nanosheets have ordered mesoporous structure, high crystalline anatase nanocrystal (~6 nm), large surface area (~252 m^2^/g) and uniform mesopores (~3.4 nm), as well as excellent electrical properties that can be potentially applied to lithium-ion batteries.

**Figure 3:**
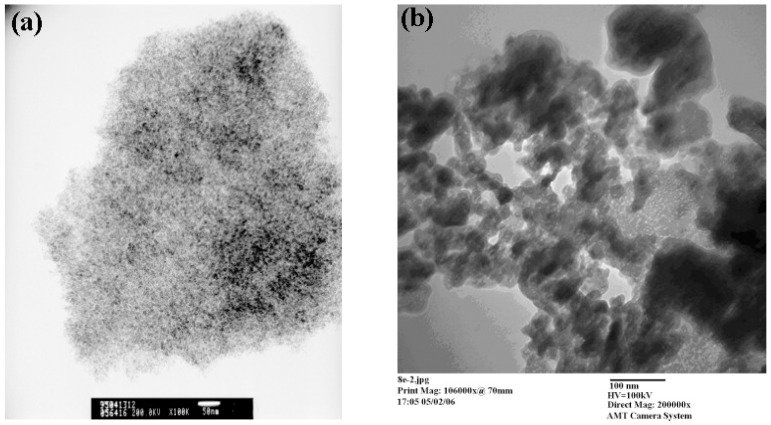
TEM image of (**a**) M-TiO_2_ and (**b**) doped M-TiO_2_. Reprinted with permission from reference [23]; Copyright 2018, Elsevier.

The large employment of sol–gel method in the preparation of mesoporous TiO_2_ has greatly expanded its application field as well. Lee and co-workers prepared a pure mesoporous TiO_2_ (M-TiO_2_) (Figure 3a) and a W-doped mesoporous TiO_2_ (doped M-TiO_2_) using sol–gel method (Figure 3b) [23]. The presence of W in doped M-TiO_2_ decreases the energy gap of the material, which shifts the optically responsive region of TiO_2_ into the visible light range, and energy gap (Eg) through W doping is reduced to a maximum of 1.77 eV, which is considered to be the limit of W. Moreover, differences in the content of W also affects the anatase crystal size. The synthesized doped M-TiO_2_ has a promising potential as visible light photocatalyst.

### 2.2. Hydrothermal Method

**Figure 4:**
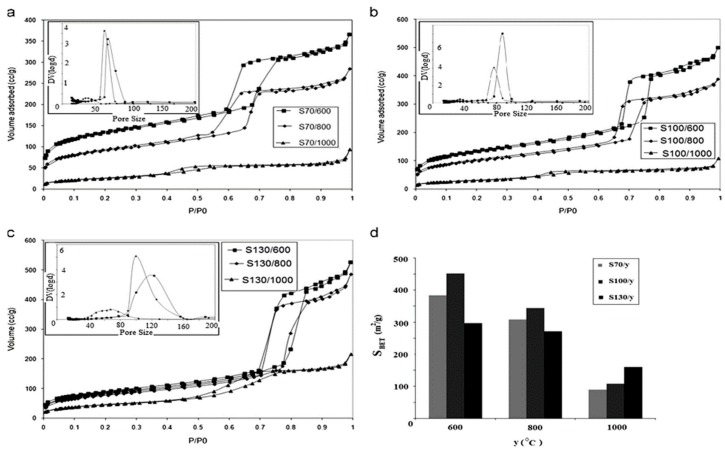
(**a**–**c**) BJH (Barrett–Joyner–Halenda) pore size distribution of the samples under same reaction temperature and different calcination temperature and (**d**) the relationship between calcination temperature and samples surface area. Reprinted with permission from reference [24]. Copyright 2018.

Hydrothermal method is very useful in preparation of microporous and mesoporous TiO_2_ with a direct and great controlling of the shape. The method involves reaction under high temperature and pressure conditions in a sealed pressure vessel, using water as the solvent. The temperature range is usually between 130 and 250 °C, and the corresponding vapor pressure of water is 0.3–4 MPa. The process does not require high-temperature calcining to prepare crystalline material, and can control the product with certain pore size and morphology by changing the hydrothermal conditions. Because of this, it is one of the preferred methods for preparing well-crystallized, non-agglomerated mesoporous materials. Compared to the template methods, the hydrothermal method is simpler because there is no post-processing for removing the organic template residues. However, the reaction factors—such as the temperature, pH and time—will have a great impact on the pore volume and shape of materials. For example, Babak and his coworkers demonstrated that mesoporous TiO_2_ with lower surface area (90 m^2^/g) and higher degree of crystallinity was prepared by controlling the hydrothermal and calcination temperatures (Figure 4) [24]. They concluded that this was due to the growth of TiO_2_ particles inside the formed nanorod/nanotube channels and the collapse of pores at higher calcination temperatures. However, when the calcination temperature of the material reached 1000 °C, the surface area and photocatalytic performance of the material would drop sharply because of the orderly loss of the mesostructure and the increase of the ratio of rutile phase.

### 2.3. Solvothermal Method

**Figure 5:**
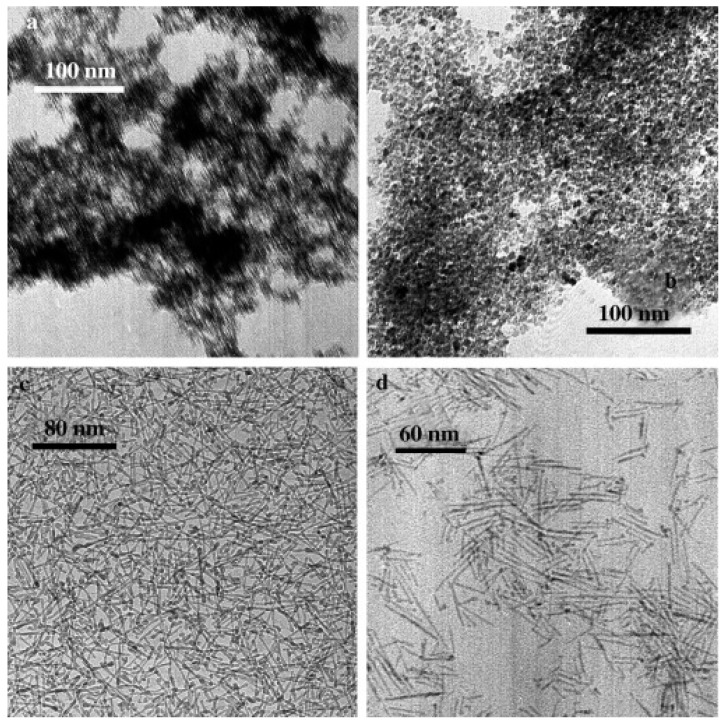
TEM images of TiO_2_ synthesized from different LA amounts: (**a**) 0, (**b**) 2, (**c**) 7 and (**d**) 25 mL. Reproduced from reference [25] with permission of Wiley Online Library.

The solvothermal method is not much different from the hydrothermal analogue except for the solvent used in the reaction. Hydrothermal method uses water as the solvent, so the reaction temperature is always lower than the supercritical temperature of water (374 °C). In contrast, in a solvothermal reaction, the temperature can be much higher since the solvent used often has a high boiling point. Unlike the hydrothermal method where the hydrolysis reaction is too fast, solvothermal reaction is generally more mild in the preparation of mesoporous TiO_2_, and thus has better control over the pore size and crystallinity. Because of this, it is often used to synthesize mesoporous materials with controlled size and high crystallinity. Li et al. demonstrated that proper controlling of the hydrolysis condition in the solvothermal process using linoleic acid (LA) as the solvent leads to higher crystallization (crystalline nanorods of 50 nm in length) of TiO_2_ [25]. The lattice fringes of nanorods corresponds to the (101) plane of anatase TiO_2_. They found that LA not only can be used as a hydrolytic reagent for reaction with titanium precursor, but also a surfactant to promote anisotropic crystal growth of TiO_2_. As the LA content increases, TiO_2_ changes from amorphous to aggregated particles then crystalline nanorods. The crystallinity of TiO_2_ will increase as the LA content keeps increasing (Figure 5).

The reaction factors of a solvothermal process, including but are not limited to the solvent type, temperatures, time, and pH, have an influence on the morphology, pore size and crystallinity of the prepared TiO_2_. For example, the pore size and volume of TiO_2_ could be altered by varying the concentration of H_3_PO_4_ hence the pH of the reaction solution (Table 2) [26]. Similarly, organic solvent was also found to affect the crystallinity and morphology of mesoporous TiO_2_ by reducing the solubility of TiO_2_ as well as by limiting the dehydration, thus forming smaller nanoparticles [27,28].

### 2.4. Template Method

**Figure 6:**
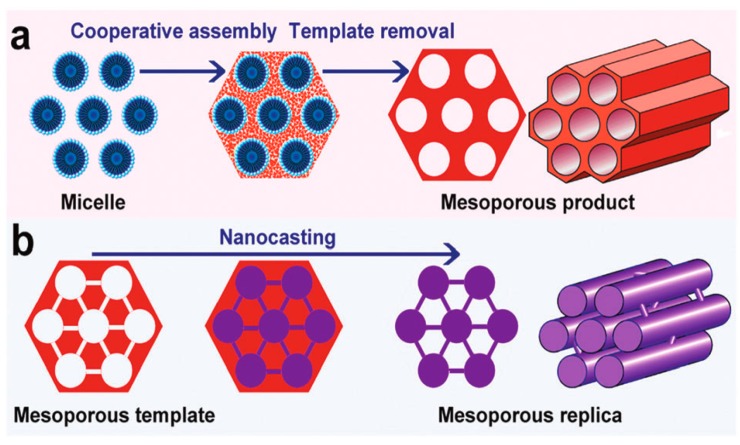
Template method for synthesis of mesoporous materials: (**a**) soft-templating method, (**b**) hard-templating (nanocasting) method. Reproduced from [29] with permission of The Royal Society of Chemistry.

Template method is usually used to synthesize well-controlled ordered mesoporous TiO_2_ because it can control the size, morphology and structure of synthesized nanomaterials based on the spatial limitation of the template with its intrinsic characteristics. Generally, template method includes hard-templating and soft-templating approaches (Figure 6) [29].

#### 2.4.1. Soft-Templating Method

Soft templates are often aggregated with surfactant molecules, including the various ordered polymers of amphipathic molecules and the self-assembly of biomolecules and polymers. Ordered mesoporous structure can be obtained by the cooperative assembly of precursors and surfactant templates in the process of soft-templating. Soft-template methods usually include syntheses in both aqueous and non-aqueous solution. The non-aqueous solution synthesis is also called evaporation induced self-assembly (EISA) method. The organic molecule templates are critical for mesostructures because of their constituents and properties, therefore they are also known as structure-directing agents (SDA) [16,29]. The molecule templates for the preparation of mesoporous TiO_2_ are normally consisting of non-ionic surfactants (such as Pluronic P123, F127) and ionic surfactants (such as CTAB, SDS) [30,31,32,33,34,35,36]. For example, in 1995, the first reported mesoporous TiO_2_ was synthesized by Antonelli and Ying via an aqueous soft-templating route [3]. In this process, titanium acetylacetonate tris-isopropoxide was employed as a precursor. A mesostructured TiO_2_ was obtained with a phosphate surfactant in acidic condition after ageing at 80 °C for two days. After calcination at 500 °C, the ordered mesoporous TiO_2_ was obtained with surface area of 200 m^2^·g^−1^ and pore size of 3.2 nm, a typical value for mesoporous materials. Since then, the method has been widely used to prepare various mesoporous TiO_2_.

Since titanium precursors are very reactive and also very sensitive to moisture, so for the aqueous solution route, the hydrolysis is normally too fast to control so the polymerization is very difficult. For instance, Ti alkoxide is nearly five orders of magnitude faster than the silicon alkoxide in terms of hydrolysis rate. The resultant mesoporous TiO_2_ generally does not have a high degree of structural order. Therefore, the key in the preparation of ordered mesoporous TiO_2_ with soft-template method is to regulate the hydrolysis and condensation rate of Ti precursors and the synergistic assembly with surfactants.

**Figure 7:**
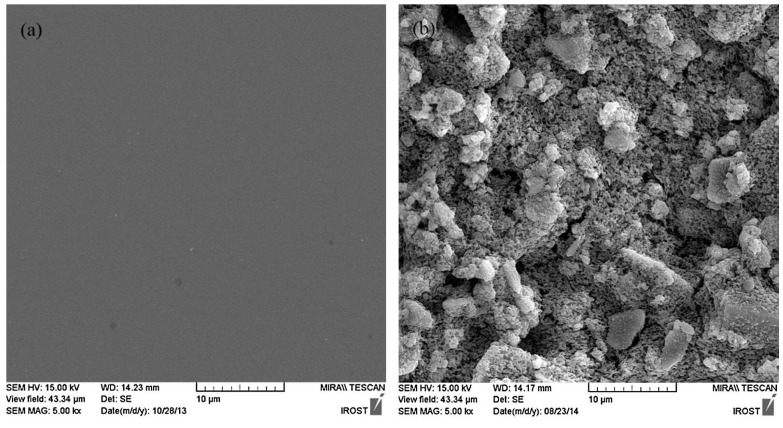
FE-SEM images of (**a**) TiO_2_ films and (**b**) mesoporous TiO_2_ films. Reprinted with permission from reference [38]. Copyright 2018, Elsevier.

In comparison, the EISA route can effectively slow down the hydrolysis and condensation rate of the Ti precursor by using a non-aqueous solvent medium. This method is conducive to the formation of ordered mesoporous structure, which can be used to prepare ordered mesoporous TiO_2_ film and powder [30,37]. For example, Mohamad et al. synthesized mesoporous TiO_2_ films and powders by combining sol–gel and EISA methods. These composite films exhibited a mesoporous non-cracked morphology with a thickness of about 300 nm (Figure 7) [38]. The synthesized powders, including a fusion of anatase and rutile phases, had a surface area of 114 m^2^·g^−1^ and a pore size of 5.8 nm.

**Scheme 1:**
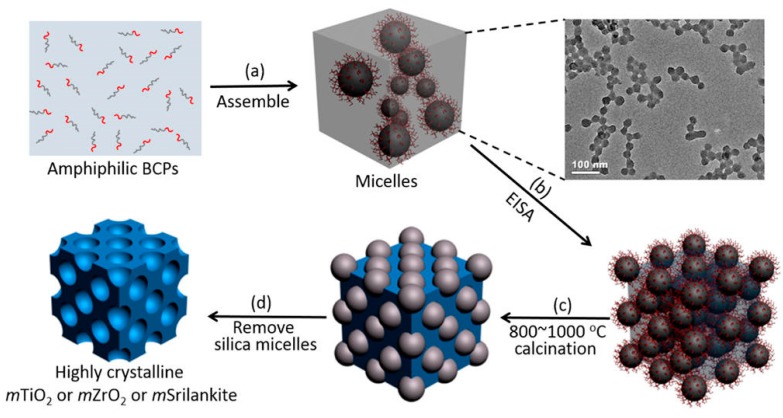
Synthetic diagram of highly crystalline mesoporous materials from colloidal amphiphilic-templating approach. Reprinted with permission from [39]. Copyright 2018, American Chemical Society.

Recently, He and coworkers synthesized highly crystalline mesoporous TiO_2_ using colloidal amphiphile (CAM)-templating, where CAM consists of polymer chains and nanoparticles (Scheme 1) [39]. In a typical process of such synthesis, CAMs composed of nanostructure TiO_2_ nanoparticles and PEO hydrophilic tethers are used as structure-directing agents. This CAM-templating method has the advantages of both the soft template method and the hard template method. Compared to other soft templates with polymers of amphipathic molecules, CAMs have excellent thermal stability and mechanical strength as well as the ability to control crystalline phase transition. In addition, the product can straightly crystallize under the calcination temperature of 1000 °C, and its ordered mesostructure will not be destroyed. The synthesis strategy with CAMs as soft templates can be used to construct a variety of highly crystalline mesoporous materials.

#### 2.4.2. Hard-Templating Method

The hard-templating method is similar to the traditional casting method in its entire manufacturing process, only that the hard-templating is on the nanometer scale so is also called nanocasting method (Figure 6b). Hard-template uses covalent bond to maintain a specific shape of the template, such as polymers with different spatial structures, carbon nanotubes, metals, and minerals. The preparation generally involves the following steps: (i) the precursor fills into the interior of the template; (ii) morphology is copied to the target product; and (iii) template is removed by acid–base dissolution and pyrolysis. Although the hard template method is a little more complex than the soft template method, it has advantages that some soft template methods do not have, such as the ability to avoid the effects of hydrolysis and polycondensation rates of Ti precursors, and co-assembly with surfactants. It can also prevent the destruction of TiO_2_ frames in intermediary pores during phase transformation. Therefore, the synthesized ordered mesoporous TiO_2_ usually has a novel mesoporous structure as well as excellent thermal stability and crystallinity [16].

**Figure 8:**
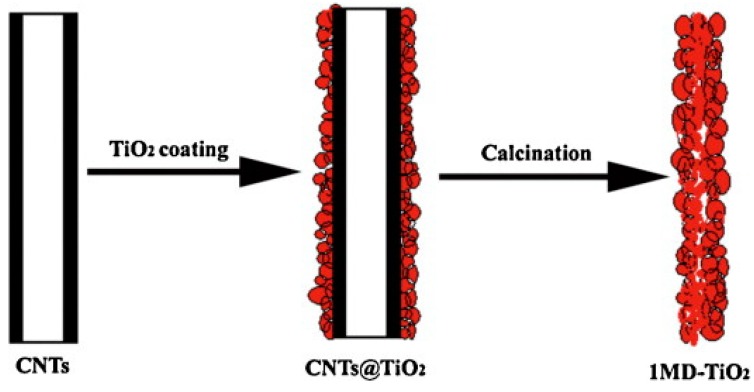
The fabrication process of 1DM-TiO_2_. Reprinted with permission from reference [40]. Copyright 2018, Elsevier.

**Figure 9:**
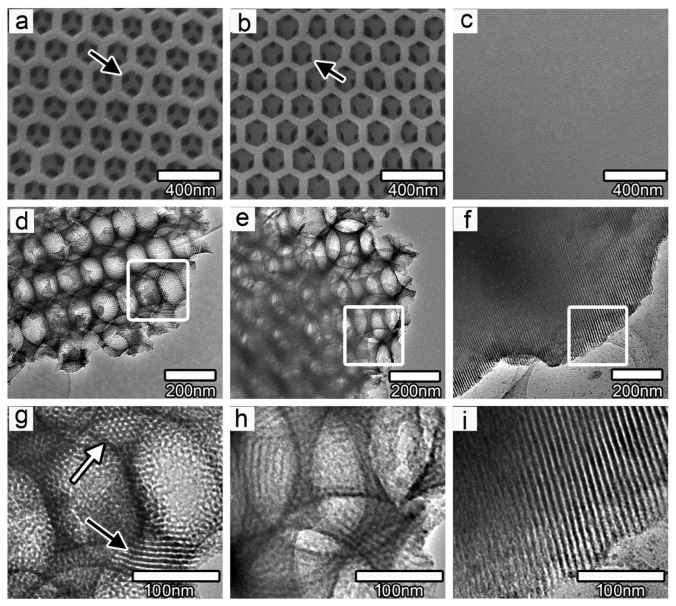
TEM and SEM images of (**a**,**d**,**g**) macro-mesoporous TiO_2_ films and (**b**,**e**,**h**) with graphene and (**c**,**f**,**i**) pure mesoporous TiO_2_ film. (**a**,**b**) The black arrows indicate the interconnected channels between macropores in the film, while (**g**) the white and black arrows point the mesopores contained in the macropore walls. Reprinted with permission from [41]. Copyright 2018, American Chemical Society.

The hard templates used in this method generally include nano-sized particles, colloidal crystals, and mesoporous materials. In the hard-template procedure, ordered mesoporous TiO_2_ materials can be obtained using these highly ordered mesoporous materials as templates. For example, nanocrystals constituted one-dimensional (1D) mesoporous anatase TiO_2_ was successfully synthesized using carbon nanotubes (CNTs) as the template (Figure 8) [40]. The product has nano-scale and submicro-scale components, as well as porous structures. In addition, the synthesized mesoporous TiO_2_ has one-dimensional mesoporous structure, nanocrystals and large surface area (102.1 m^2^·g^−1^) and pore size (12 nm), which provides good lithium storage capacity and excellent cycle performance that can be well applied in lithium batteries. Moreover, Du et al. synthesized two-dimensional (2D) hexagonal (p6mm) ordered mesoporous TiO_2_ by employing colloidal crystals as a template [41]. The synthesized ordered mesoporous TiO_2_ had not only interconnected periodic macropores, but also large surface area and pore size with 256 m^2^·g^−1^ and 4.9 nm, respectively. The presence of these interconnected macropores decreases the length of the mesoporous channel and enhances the specific surface area of the material (Figure 9). They also added graphene to the ordered macro-mesoporous TiO_2_ structure. Due to its unique electrical properties, graphene effectively inhibits the recombination of charge in the film. Therefore, this composite film enhances the rapid adsorption and photodegradation of organic dyes that can be applied in the removal of organic pollutants in wastewater treatment and air purification.

There are some differences in the resultant products and the processes for the four synthesis methods. For the hydrothermal method, the process is simple and the purity of resultant product is high, but its structure is irregular, because the hydrolysis rate of titanium precursor is too fast to control the morphology of the target product. At the same time, the dispersion of particles is poor and the accumulation is very serious; For solvothermal method, the hydrolysis rate of Ti precursor is generally more mild, and pore size and crystallinity can be better controlled, but the purity is poor because the solubility of the precursor in organic solvents is limited. Template method is usually used to synthesize mesoporous TiO_2_ and other nanomaterials with well-controlled ordered structure, because the size, morphology and structure can be controlled by this method based on the spatial limitation of the template with its intrinsic characteristics. However, the final step in the template method is generally the removal of the template, which sometimes causes collapse of mesoporous structure and long process time. Sol–gel method is the most common and versatile way for preparing mesoporous TiO_2_ with good uniformity and high purity, but the product often cannot be calcined at high temperatures. Therefore, this method is often used in combination with the template method.

### 2.5. Morphology

Over the past two decades, many morphologies of mesoporous TiO_2_, including mesoporous spheres, nanotubes and films, have been prepared by different methods. Varying the synthesis methods, as discussed in previous sections, may result in completely different morphologies for the final products. Whereas the morphologies of mesoporous TiO_2_ synthesized by various methods are closely related to their applications. In other words, the morphology of mesoporous TiO_2_ largely determines its applications, for example, mesoporous TiO_2_ spheres were often used as photocatalysts for pollutant degradation. The preparation of spheres and hollow spheres usually undergoes a sol–gel process. Morphology of these materials have highly porous structure and large surface area, which is beneficial for various applications including as photocatalysts and in energy and environment related fields. Mesoporous TiO_2_ spheres may be disordered or ordered in pore size structure, where the ordered ones include scopes from micropores to macropores.

Mesoporous TiO_2_ nanoparticles usually exist in the form of nanotubes, nanorods, nanofibers, nanowires and nanosheets. These materials are usually synthesized by sol–gel or hydrothermal method [42, 43,44]. Because of the specific surface area and crystallinity, nanoparticles are usually used to improve the photo-electron activity of the mesoporous TiO_2_. For example, Guo et al. synthesized nanostructured mesoporous TiO_2_ with superior electrode performance [42]. The nano-sized network can reduce the diffusion time of electrons and enhance the local conductivities, hence it has excellent power properties and can be applied to the field of lithium batteries. Recently, the nanostructure of hierarchical V_2_O_5_ nanorods on TiO_2_ nanofibers is prepared by Ghosh et al. [43]. The fabrication of hierarchical ‘nanorods-on-nanofiber’ heterostructures undergoes gas jet fiber (GJF) spinning process. The heterostructure V_2_O_5_-TiO_2_ materials have higher photocatalytic activity due to the slowdown of electron-hole charge recombination in heterostructures, which can be used as an excellent photocatalyst. In addition, Ghosh et al. also successfully synthesized bi-component mesoporous TiO_2_–ITO (indium tin oxide) nanofibers with core-shell (CS) and side-by-side (SBS) structure by GJF spinning process [44]. At a calcination temperature of 700 °C, the CS and SBS nanofibers had a specific surface area of 19.9 and 15.5 m^2^/g, respectively, and exhibited the presence of anatase and rutile phases of TiO_2_ and cubic crystals for ITO. This research provided an effective pathway for the synthesis of mesoporous nanofibers of multiple morphologies.

**Figure 10:**
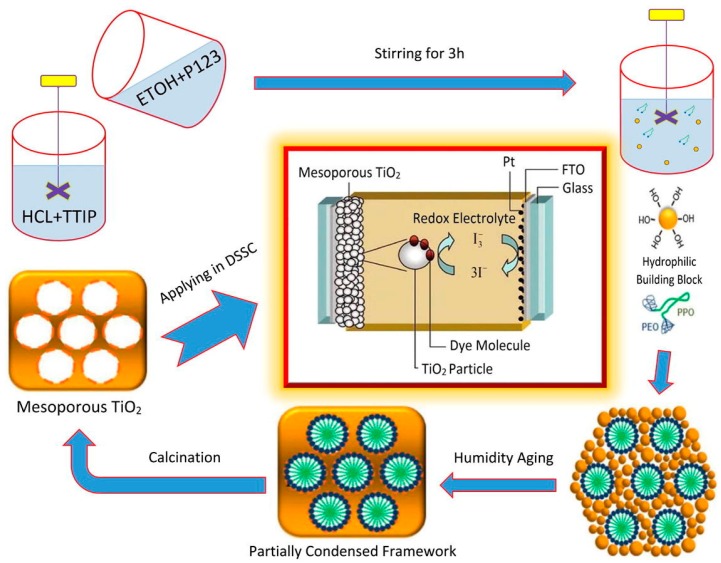
Schematic formation process of the mesoporous TiO_2_ via EISA. Reprinted with permission from [38]. Copyright 2018, Elsevier.

Mesoporous TiO_2_ films with rough surface in connected pores favor the mass transport and therefore have wide applications in drug delivery and composite photocatalysts. Mesoporous TiO_2_ films are usually prepared by the template methods. EISA method has also been successfully employed to synthesize mesoporous TiO_2_ films with thickness of 250–700 nm and pore size of 4–7 nm. Mohamad et al. combined sol–gel and EISA method to prepared mesoporous TiO_2_ films using triblock P123 template [38]. The films with thickness of 300 nm were synthesized from TiO_2_ sol (the molar ratios of TTIP:HCl:EtOH:P123 = 1:1.6:26:0.01), then aged under 10% relative humidity and at the 5 °C for 72 h (Figure 10). Li et al. synthesized mesoporous TiO_2_/SiO_2_ hybrid films by the polymeric micelle-assembly method [45]. They used a triblock copolymer, poly(styrene-b-2-vinyl pyridine-b-ethylene oxide), as a template. The process involved an effective interaction of TTIP and TEOS with the polymeric micelles temple, and then the mesoporous films was obtained by calcination at different temperatures (450, 550 and 650 °C).

### 2.6. Crystallization

There are four main crystalline forms of TiO_2_ in nature: anatase, rutile, brookite and TiO_2_ (B). Some of the most important parameters of TiO_2_ are summarized in Table 3 [46,47]. Anatase has higher photocatalytic activity because of containing more defects and vacancies, which can produce more oxygen vacancies to capture electrons. Rutile is the most stable crystalline structure with less defects, and this leads to the easy recombination of electrons and holes, hence it has poor photocatalytic activity as compared to anatase. While brookite is the most unstable crystal form, it will be converted to rutile when the temperature is higher than 650 °C [46]. TiO_2_ (B) is the least dense of four crystalline forms of titania.

Amorphous or semicrystalline TiO_2_ usually includes large numbers of defects, which cause the easy recombination of electrons and holes, therefore high crystalline structure TiO_2_ (such as anatase and brookite phase) with fewer defects is always required to improve the conversion efficiency of solar energy. The porous structure (such as high surface and ordered mesopore channel, etc.) of TiO_2_ is also an important factor that can enhance the light scattering and dye absorption. The crystallinity of the mesoporous TiO_2_ is closely related to the calcination temperature. High crystallinity generally requires a high calcination temperature. When the calcination temperature is too high, however, it sometimes causes the collapse of mesoporous structure [11,24]. Therefore, the calcination temperature is usually lower than 450 °C when the high crystallinity mesoporous TiO_2_ is synthesized by the soft-templating method. For the hard-templating method, the mesoporous structure can still be maintained under higher calcination temperature, but this method still has some shortcomings, such as long process time and high cost.

### 2.7. Doping

Although mesoporous TiO_2_ photocatalysts have been widely used, certain disadvantages, for example low photonutilization efficiency and poor absorption of visible light due to large band gap of TiO_2_ (3–3.2 eV) have also limited their application. Therefore, a large amount of efforts has been directed to the modification of the material with the purpose of improving the photocatalytic effect and activity of mesoporous TiO_2_ photocatalysts [48,49,50,51,52]. Among them, doping is one of the most promising ways for enhancing the performance of mesoporous TiO_2_. Not only can the TiO_2_ bandgap energy be reduced, but also the charge separation efficiency of TiO_2_ can be improved by this strategy [9,11]. Generally, doping includes non-metal doping, metal doping and co-doping.

Doping of non-metal elements (e.g., C, N, S, etc.) on mesoporous TiO_2_ can widen its valence band and narrow the bandgap so that the surface is more likely to generate highly active electrons and holes, which makes the photocatalysis more effective [53,54,55,56,57,58]. During the doping process, the non-metal anions will replace the lattice oxygen anions in TiO_2_ and present as isolated atoms. Francesco and co-workers synthesized a carbon-doped mesoporous TiO_2_ photocatalyst with excellent activity that can be employed to degrade phenolic compound and caffeic acid under visible light [55]. This is attributed to the doping of C, which shifts the optical responsive spectrum of TiO_2_ to the visible range. Likewise, Mohamad et al. synthesized a C-doped mesoporous rutile TiO_2_ by bio-template assisted sol–gel method [56]. The presence of carbon in the product provided a synergistic effect and promoted higher photocatalytic activity that greatly enhanced the absorption of visible light as well.

In contrast with non-metal doping, doping of metal elements (such as Fe, Ag, Ce, La, etc.) on mesoporous TiO_2_ can also reduce the band gap width and change the photoresponsive area [59,60,61,62,63]. For example, Xiang et al. formed anatase TiO_2_/Ag heterostructure films via a facile electrochemical approach [61]. Compared with bare TiO_2_ films, Ag-doped mesoporous TiO_2_ films significantly improved the degradation of methylene blue in the UV (ultraviolet) and visible light regions. Recently, magnetic cerium-doped mesoporous TiO_2_ was prepared by Mermana and co-workers [62]. The as-prepared photocatalyst possessed high photocatalytic degradation efficiency, which can be reused at least three times. The study also demonstrated the promising future application in environment-related fields of photocatalytic degradation of mesoporous TiO_2_.

Single element doping of mesoporous TiO_2_ can only increase the valence band energy of TiO_2_ or decreases its conduction band energy, thus reducing the bandgap width of TiO_2_. In contrast, co-doping can overcome the limitations of a single doping, where the valence band energy and conduction band energy can be altered simultaneously. Some work regarding the co-doping of mesoporous TiO_2_ was reported in recent years [64,65,66,67,68,69]. This includes metal–metal co-doping, non-metal–non-metal co-doping and non-metal–metal co-doping. Zhang and workmates used a fast sol–gel method to prepare Fe-N co-doped mesoporous TiO_2_ photocatalyst [65]. The co-doped products showed higher photocatalytic activity, larger surface area and more ordered mesoporous structure, as compared to pure and single doping TiO_2_ products. A study also confirmed that co-doping can reduce the bandgap of TiO_2_, where the band gap of co-doped mesoporous TiO_2_ was found between 2.71 eV and 2.76 eV [67].

Despite doping in mesoporous TiO_2_ achieving great success, several challenges remain: (i) how to control the spatial distribution of dopant-induced electron states and surface heteroatoms or heterostructures to promote charge carrier transfer over the surface of mesoporous TiO_2_; (ii) how to make dopants evenly distributed in mesoporous TiO_2_ systems; (iii) how to maintain the cavity structure of the mesoporous TiO_2_, as well as the high specific surface area, pore size and crystallinity.

The commonly used Ti precursors for the fabrication of mesoporous TiO_2_ materials, as well as the related parameters of the corresponding synthesis materials are summarized in the Table 4.

## 3. Application

Mesoporous TiO_2_ as a class of functional materials has been intensively studied for their promising technological applications, particularly those in photocatalysis, biology and energy. Herein, we focus on the latest work of its application in these fields.

### 3.1. Photocatalysis

Nowadays, advanced photocatalysis semiconductors have received extensive attention in the environmental fields because of their huge potential, for example in the removal of organic pollutants, and to deodorize and purify polluted air and water as well as used for solar water splitting [79,80,81,82,83,84,85,86,87,88,89,90]. Among many studied photocatalysis semiconductors, mesoporous TiO_2_ is a one of the most preferable materials because of its continuous particle framework, high quantum efficiency, high surface area, nanocrystallinity, long-term stability and non-toxicity.

The pollution caused by coloring and textile industry has been a significant environmental concern. The colors and dyes produced by these industries are organic compounds that are not biodegradable. Most of these organic contaminants, however, can degrade through photocatalysis under ultraviolet or visible-light irradiation with mesoporous TiO_2_ as the catalyst. These include bisphenol A, methyl orange, Rhodamine B, etc. Recently, Chaker et al. prepared a silver-doped mesoporous TiO_2_ that has excellent catalytic degradation ability for methyl orange and wastewater under ultraviolet and simulated solar light irradiation. Compared to P25 TiO_2_, the prepared doping material has a strong ability to restrain e^−^-h^+^ (photogenerated electron hole pair) recombination that helps increase the photocatalytic activity [81]. Zul et al. synthesized a polyethersulfone-TiO_2_ (PES/TiO_2_) film photocatalyst that is also very efficient in the decomposition of methyl orange [82]. The film was found to have very good stability that can be used in both acidic and basic conditions, and can maintain high degradation efficiency after five cycles.

**Figure 11:**
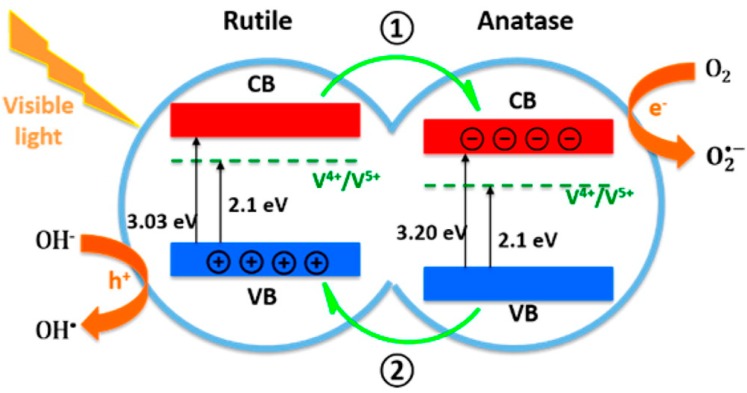
Schematic illustration of the synergistic mechanism of rutile and anatase. Reprinted with permission from [87]. Copyright 2018, American Chemical Society.

It is well known that commercial TiO_2_ (Degussa P25) photocatalysts are often used as a measure of photocatalytic performance of the prepared products due to its good photocatalytic activity. However, it still has some drawbacks in pollutant degradation because of its nonporous structure, relatively low surface area and visible light photocatalytic activity. Recently, Luo et al. synthesized a mesoporous TiO_2_ containing anatase/rutile mixture phase [87]. The research had shown that the presence of mixed phases can increase the photocatalytic activity. Because the energy gap of the V^4+^/V^5+^ impurity in the mixed phase can be excited by visible light (Figure 11), the products are shown to have much better photocatalytic activity than Degussa P25 after visible light irradiation. Moreover, the results indicated that methylene blue dye (MB) degradation can achieve an efficiency of as high as 100%. Ghosh et al. also testified that the presence of mixed phases of mesoporous TiO_2_ can increase the photocatalytic activity [74]. The prepared mesoporous TiO_2_ nanofibers (36% rutile phase and 64% anatase phase) showed higher photocatalytic activity and had the ability of purifying indoor air pollution caused by volatile organic compounds, which can decompose ethanol into CO_2_ and H_2_O without the intermediate products of acetaldehyde and formic acid.

Hydrogen is an ideal clean fuel. Photocatalytic water splitting using solar energy to produce hydrogen is considered to be a promising strategy for solving energy and environmental problems. While mesoporous TiO_2_ deposited with plasmonic metal nanoparticles (Ag, Au, Pt, etc.) have been extensively studied for solar water splitting, and the presence of these nanoparticles can improve the photocatalytic efficiency of TiO_2_. For example, Shang et al. synthesized Ag@TiO_2_ mesoporous nanofibers for the photodecomposition of water [91]. The results indicated that incorporation of Ag nanoparticles into mesoporous anatase TiO_2_ nanofibers significantly improved their photocatalytic efficiency. This work provided potential applications for efficient hydrogen evolution. Atabaev et al. prepared mesoporous rutile TiO_2_ deposited with Pt nanoparticles for photocatalytic water splitting [92]. The results also showed that the enhanced photocatalytic efficiency is due to the presence of Pt in the resultant.

### 3.2. Solar Cells

There is no doubt that solar energy is a rich, clean and renewable energy source. At present, the study of solar energy has been extensively advanced. Mesoporous TiO_2_ materials have been shown to have high conversion efficiency and can therefore be used as solar cells. In this section, mesoporous TiO_2_ utilized in dye-sensitized cells (DSSCs) and perovskite solar cells are discussed.

DSSCs are solar cells that use low-cost mesoporous TiO_2_ and photosensitive dyes as the main raw material to simulate the use of solar energy of the plants in nature in the form of photosynthesis that transforms the solar energy to electricity. A typical DSSC consists of nanoporous semiconductor films, dye sensitizers, redox electrolytes, counter electrodes and conductive substrates. Thanks to its large surface area and high crystallinity, ordered mesoporous TiO_2_ will improve the conversion efficiency of DSSCs by enhancing the dye diffusion and adsorption, as well as the electron transport efficiency [93,94]. Different morphology in the mesoporous TiO_2_ makes a difference in the conversion efficiency of resultant DSSCs.

**Figure 12:**
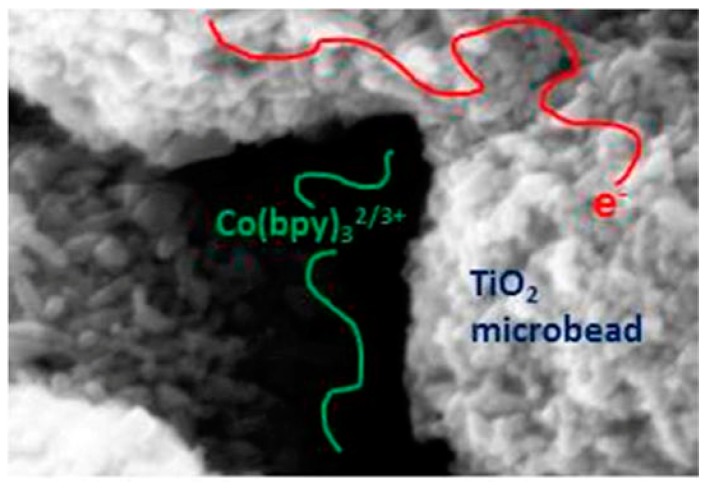
Diffusion in mesoporous mircobeads. Reprinted with permission from [95]. Copyright 2018, American Chemical Society.

There are several studies about the role of porous structures and morphology of TiO_2_ in improving the conversion efficiency of DSSCs and enhancing the light scattering and dye absorption [95,96,97,98]. Meysam et al. synthesized TiO_2_ mesoporous microbeads by combining sol–gel and solvothermal methods [95]. This light scattering microbead film achieved a photoelectric conversion efficiency of 6.4% with an electron transport rate that is significantly faster than the standard mesoporous TiO_2_ film. This improvement resulted from the good diffusion of electrons in the microbead film (Figure 12). Hou et al. prepared a mesoporous TiO_2_ using polyvinyl pyrrolidone (PVP) as a film and pore generator [96]. They found that porous TiO_2_ electrode with highly specific surface area play a significant role in DSSCs, which can absorb a great deal of dye and cause the electrolyte to penetrate into TiO_2_ pores. The DSSC of mesoporous TiO_2_ with PVP (the surface area with 89.50 m^2^·g^−1^) yielded a higher conversion efficiency of 8.39%, which is about 57% higher than that without PVP (the surface area with 69.91 m^2^·g^−1^). This is because the removal of PVP forms interconnected pores inside TiO_2_, therefore enhancing the surface area for dye adsorption and effective electron transport.

Though DSSCs have reached a higher conversion efficiency, they also faced some challenges, such as the preparation of high-efficiency electrodes under low-temperature, the development of inexpensive and stable full-spectrum dyes and sealing of liquid electrolytes, etc. In contrast with DSSCs, CH_3_NH_3_PbI_3_ perovskite solar cells based on a mesoporous TiO_2_ scaffold possess all conditions for a perfect light-absorbing material: a suitable direct bandgap, a high absorption coefficient, excellent carrier transport properties and a high defect tolerance. These allow perovskite solar cells to have a relatively higher energy conversion efficiency. Mesoporous TiO_2_ is frequently used as the electron transport layer in the perovskite solar cells. Presently, perovskite solar cells are the most studied class of solar cells [99,100,101,102,103,104]. For example, Zhu and co-workers reported about a perovskite solar cell by inserting an ultrathin graphene quantum dots (GQDs) layer between perovskite and mesoporous TiO_2_. The efficiency of this solar cell (>10%) is significantly higher than that without GQDs (8.81%), which is mainly attributed to the boosting of the photocurrent [102].

### 3.3. Lithium-Ion Batteries

Although TiO_2_ has the advantages of small volumetric strain when intercalated with lithium, high lithium insertion potential and no solid passivation film, the low electronic conductivity of TiO_2_ prevents it from a good lithium-ion battery material. TiO_2_ may accommodate up to one lithium formulation unit to form Li_*x*_TiO_2_, which limits the diffusion of lithium ions and conduction of electrons, therefore decreases the cycle stability and capacity of materials. TiO_2_ materials with mesoporous structure, however, have better electrical conductivity and lithium ion diffusion properties. The mesoporous structure is not only conducive to the electrode surface contact with the electrolyte to improve the battery output power, but also can shorten the lithium ion from the electrode surface into the electrode within the required diffusion distance and make it easier to insert/pull out the lithium-ion electrode. In addition, the morphology of the mesoporous structure also affects the electrical properties of the material.

At the present time, mesoporous TiO_2_ with different morphologies (such as hollow microsphere, nanosheet, nanotube, sub-microsphere, etc.) have been widely used as electrodes in lithium-ion batteries. For example, Tian and colleagues synthesized mesoporous TiO_2_/SnO_2_/C hollow microspheres that were used as the anode of lithium-ion batteries [105]. This synthetic mesoporous material had excellent cycling stability in a lithium-ion battery. Because of the nanostructures present in the system, volume changes and structural stresses are greatly reduced, electron and ion transport are largely increased, therefore excellent cycling stability is reached. Recently, Sun and co-workers synthesized mesoporous TiO_2_ sub-microsphere for lithium-ion battery [106]. The resultant product has an excellent porous structure. The pore volume can reach 0.792 cm^3^·g^−1^, while the specific surface area can achieve 291.08 m^2^·g^−1^. Moreover, this lithium-ion battery also has a higher specific capacity and a longer cycle life.

### 3.4. Biological Applications

#### 3.4.1. Biosensors

A biosensor is a small device that responds to biological substance and converts to electrical signals for detection. Biosensors have the advantages of high sensitivity, short response time, superior stability, and excellent biocompatibility. Mesoporous TiO_2_ materials not only have large surface area and uniform pore size distribution, but are also biocompatible and environmentally-friendly, which makes them promising biosensors. Wu et al. synthesized a ε-poly-l-lysine-modified mesoporous TiO_2_ that was used in biosensors for immobilizing negatively charged enzymes [107,108]. By proper modifications, the mesoporous TiO_2_ surface can form multilayers and complex structures, which gives the immobilized enzyme high operational stability, storage stability, thermal stability and good reusability, which are often considered as the most important biological parameters of biosensors.

#### 3.4.2. Cancer Therapy

The rapid development of nanotechnology and nanomedicine has promoted the emergence of a variety of new treatments for the effective treatment of cancer. Compared to other forms of cancer treatment such as surgery, chemotherapy, radiology, and other therapies, the mild non-invasive photodynamic therapy (PDT) is a new and pioneering solution. PDT utilizes the photoexcitation of photosensitizer (PS) to generate high reactive oxygen species (ROS) that can destroy toxic cells [109,110]. As the most widely used semiconductor, mesoporous TiO_2_ has been used in PDT thanks to its low cytotoxicity, stable mesostructure, large surface areas and uniform pore size. In a PDT treatment, the mesoporous TiO_2_ mainly works as a photosensitizer. Yu et al. reported a novel TiO_2_-coated Fe_3_O_4_ nanoparticle core/shell nanocarriers (Fe_3_O_4_@TiO_2_@mTiO_2_) [111]. The material can be used as a photosensitizer for photodynamic therapy (PDT) under NIR light. The prepared photosensitizer was used to carry DOX (doxorubicin hydrochloride) chemotherapy drug and β-catenin siRNA. The study shows a combined medical treatment and diagnosis feature of the complex TiO_2_ system that leads to a high specificity and anti-tumor efficacy and has provided a new way to the imaging-guided cancer treatment.

In addition to PDT, another new method, namely sonodynamic therapy (SDT), is also a very effective cancer treatment [112,113,114]. Compared with PDT, SDT can reduce the risk of light penetrating of PDT, because it utilizes ultrasound (US) to activate the somatosensory agent to produce ROS, and induces tumor death and inhibits tumor growth. Recently, Wang et al. synthesized mesoporous anatase TiO_2_ nanoparticles (MTNs) with single-crystalline structure [115]. They found that the MTN-assisted SDT can obviously enhance the tumor growth inhibition effect. This work paves a new way for cancer therapy based on mesoporous TiO_2_.

The specific applications of mesoporous TiO_2_ materials in the biological fields are summarized in Table 5.

## 4. Conclusions and Perspectives

As a commonly used filler, mesoporous titanium dioxides have received huge research attention. Compared with regular TiO_2_, the mesoporous TiO_2_ has some specific properties with good chemical and physical properties, non-toxicity and good biocompatibility, excellent photoelectric performance. Preparation including sol–gel, hydrothermal, solvothermal method and template methods were developed rapidly over the past two decades. Among the methods discussed herein, sol–gel processes are the simplest, most cost-effective method and can produce high purity products because of their availability, easy-handling, whereas templating method is often used where highly ordered structures are required for mesoporous TiO_2_. More specifically in templating methods, controlling the hydrolysis and condensation rate of Ti precursors is the key to obtaining ordered mesoporous TiO_2_. Various morphologies of mesoporous TiO_2_ have also been prepared by these methods, such as mesoporous spheres, nanotubes, nanofibers, nanorods, hierarchical nanorods on nanofibers and films, etc. Different morphologies of mesoporous TiO_2_ is closely related to its applications. Besides, mesoporous TiO_2_ in photocatalysis, solar cells, lithium-ion batteries and biological applications are also discussed. Doping on the mesoporous TiO_2_ materials is an effective way of improving their photocatalytic effect and activity.

Despite significant advances in the study of mesoporous TiO_2_, the use of inexpensive, low-toxicity, and reproducible methods to prepare mesoporous TiO_2_ materials with large surface area and high crystallinity remains a big challenge. Soft-templating and hard-templating approaches are generally used for the synthesis of ordered mesoporous TiO_2_, but the templates need to be removed after preparation, which leads to a long time and cost. The development of new synthesis routes, such as colloidal amphiphiles (CAMs) as soft templates, should be given more emphasis in the future, for that CAMs not only have excellent thermal stability and mechanical strength, but can also give highly crystalline mesoporous materials. Although mesoporous TiO_2_ offers exciting opportunities to solve energy and biological related issues, it still faces some serious challenges. In the field of photocatalysis, the application of TiO_2_ is limited to UV light is a major disadvantage. However, doped mesoporous TiO_2_ or TiO_2_ heterostructure can be considered for the improvement of its visible light photoactivity. In solar energy conversion application, different morphologies of TiO_2_ nanostructures should be designed to adjust their optical and electronic properties to increase energy conversion efficiency. While the preparation of mesoporous TiO_2_ partially improves the conductivity and lithium-conducting ability than ordinary TiO_2_, its electronic conductivity and lithium diffusion rate still limit its wide range of applications. Therefore, in the future, focuses should be emphasized on the development and fabrication of nanostructure composite materials, and the conductive and lithium-conducting capabilities can be enhanced by the synergistic effect of TiO_2_, thereby improving its electrochemical performance and prolonging the cycle life. In addition, mesoporous TiO_2_ has made some new appearances in biosensors and drug delivery applications, such as the use of mesoporous TiO_2_ as a carrier for photodynamic therapy (PDT) to treat cancer. Nevertheless, further works are required to advance the application to a practical level, such as the study of the biological effects of mesoporous TiO_2_ photosensitizers and the clinical application of mesoporous TiO_2_ anticancer photosensitizers, and the further study of its toxic effects in humans to obtain the best results.

## Figures and Tables

**Table 1:** Structural parameters of mesoporous TiO_2_ calcined at 400 °C. Reproduced from reference [20] with permission of Wiley Online Library.

Sample	Eg (eV)	Surface Area (m^2^·g^−1^)	Pore Size (Å)	Pore Volume (cm^3^·g^−1^)	Crystallite Size (nm)
TiO_2_	3.01	126	56	0.23	10.1
PTi 0.5%	3.08	185	34	0.23	7.3
PTi 1%	3.11	198	34	0.22	7.1
PTi 3%	3.09	269	34	0.24	5.6
PTi 5%	3.10	285	34	0.26	5.8

**Table 2:** Pore parameters of mesoporous TiO_2_ at different H_3_PO_4_ concentrations. Reprinted with permission from reference [26]. Copyright 2018, Elsevier.

H_3_PO_4_ Concentration (mol/L)	BET Surface Area (m^2^/g)	Total Pore Volume (cm^3^/g)	Average Pore Size (nm)
0.28	163	0.316	7.8
0.5	161	0.368	9.2
0.8	207	0.472	9.1
1.0	196	0.462	9.4
1.5	294	0.545	7.4
2.0	227	0.506	8.9

**Table 3:** Some parameters of the four main polymorphs of TiO_2_.

Crystal Structure	System	Density (g/cm^3^)	Band Gap (eV)
Anatase	Tetragonal	3.82–3.98	3.62
Rutile	Tetragonal	4.2–4.3	3.05
Brookite	Rhombohedral	4.1–4.2	-
TiO_2_ (B)	Monoclinic	3.6–3.8	3–3.22

**Table 4:** Synthesized mesoporous TiO_2_ by different Ti precursors.

Ti Precursor	Method	Cal. Tempt.	BET (m^2^/g)	Pore Size (nm)	Ref.
TTIP	Hydrothermal	500 °C	24–110	4–17.5	[70]
TBT	Sol–gel	550 °C	190	3.2	[71]
TTIP	Soft-template	500 °C	30.46	11.1	[72]
TiCl_4_	Hard-template	450 °C	239	4.87	[73]
TTIP	Sol–gel	500–700 °C	3.4–30	5–23	[74]
TBOT	Hydrothermal	500 °C	97.3	9.1	[75]
TiOCl_2_	Sol–gel	600 °C	38–88	4.8–19	[76]
Ti(SO_4_)_2_	Solvothermal	400 °C	>94.9	>7.0	[77]
TiCl_4_, TBOT	Soft-template	470 °C	77.77	6.60	[78]

**Table 5:** Specific biological applications of mesoporous TiO_2_ materials.

Method	Pore Size (nm)	BET (m^2^/g)	Morphology	Crystalline Structure	Application	Reference
Sol–gel	5.5	187	nanofiber	anatase	Immobilizing enzymes for biosensor	[107]
Template	6.2	-	core/shell	anatase	As nanocarrier for PDT	[110]
EISA	3.80	96.10	sphere	anatase	As sonosensitizers for SDT	[115]
Electrochemistry	30	-	nanotube	-	Implant surface modification	[116]
Sol–gel	-	-	film	rutile	Photocatalytic antibacterial agent	[117]
